# “When Relief Becomes Release: Exploring Euthanasia Through a Psychiatric Lens”

**DOI:** 10.1192/bjo.2026.11696

**Published:** 2026-06-30

**Authors:** Ahmed ibrahim, Shamim Ruhi, Mohamed abdelrazek, Mohamed Elkoshiery

**Affiliations:** 1 Norfolk and Suffolk NHS Foundation Trust, Ipswich, United Kingdom; 2 east suffolk and north essex foundation trust, Ipswich, United Kingdom

## Abstract

**Aims:**

To illustrate the heterogeneity of drivers underlying wishes to die and to consider the implications for psychiatric practice, particularly in relation to transparency, ethical formulation, and end-of-life decision-making.

**Methods:**

Four anonymised cases assessed within UK mental health services were selected for qualitative analysis. Each case involved a formal psychiatric assessment including mental state examination, capacity assessment, risk formulation, and evaluation of psychosocial context. Cases were examined thematically, with reference to the ethical principles of autonomy, beneficence, non-maleficence, and justice.

**Results:**

Case one involved a 93-year-old widower who attempted suicide following profound loneliness and bereavement. He was cognitively intact and denied depression. His distress was primarily social and existential and improved significantly with psychosocial intervention, antidepressant treatment, and relocation closer to family.

Case two described a 62-year-old woman with terminal breast cancer who retained capacity and engaged with services but concealed ongoing suicidality, later dying by suicide. Loss of autonomy and fear of dependency were prominent drivers.

Case three involved a 72-year-old woman with severe depression and Parkinson’s disease who deteriorated following her husband’s terminal diagnosis. Despite compulsory admission and maximal treatment, including electroconvulsive therapy, she showed no meaningful recovery and died shortly after discharge, raising concerns regarding proportionality and dignity.

Case four described a 67-year-old man with mild cognitive impairment whose fear of future dementia drove concealed suicidality, culminating in suicide despite minimal overt psychiatric symptoms.

Across cases, wishes to die arose from heterogeneous and often non-psychiatric drivers. In several cases, concealment of intent appeared linked to fear of coercive intervention.

**Conclusion:**

Wishes to die cannot be understood solely through diagnostic frameworks. These cases demonstrate the importance of distinguishing treatable distress from enduring suffering and recognising how legal and clinical contexts shape disclosure. Psychiatry has a central role in ethical formulation, safeguarding, and facilitating honest dialogue as end-of-life debates evolve.

